# Maternal Body Mass Index Does Not Affect Neonatal Umbilical Artery Blood Gas Parameters

**DOI:** 10.1155/2013/654817

**Published:** 2013-02-21

**Authors:** Salam E. Chalouhi, Caroline Salafia, Magdy Mikhail, Robert Hecht

**Affiliations:** ^1^Department of Obstetrics and Gynecology, Bronx-Lebanon Hospital Center and Albert Einstein College of Medicine, Bronx, NY 10457, USA; ^2^Department of Obstetrics and Gynecology, Obstetrics and Gynecology Administration, Bronx-Lebanon Hospital Center, 1650 Grand Concourse, Bronx, NY 10457, USA

## Abstract

This study was undertaken to assess the impact of obesity on fetal well-being in glucose-tolerant and nonhypertensive women. Medical charts of all patients admitted to the labor and delivery department at our institution between January, 2011 and July, 2011 were retrospectively reviewed. Patients with diabetes/impaired glucose tolerance or hypertension were excluded. A total of 100 women, 50 lean and 50 obese, were included. Umbilical artery blood gas parameters (BGPs) were compared in lean (<25 kg/m^2^) and obese (≥30 kg/m^2^) women. Obese and lean women were comparable with respect to all baseline characteristics. There was no difference in any of the BGP or Apgar scores between obese and lean patients. Pearson's correlation coefficient found no significant correlation between BMI and BGP/Apgar scores. Maternal obesity does not seem to affect BGP and fetal well-being in glucose-tolerant and nonhypertensive women.

## 1. Introduction

The prevalence of obesity has increased dramatically in recent decades both in the United States and abroad. Today, it is estimated that as many as 34% of women of reproductive age are obese (BMI ≥ 30 Kg/m^2^) [1]. Studies have consistently shown a significant association between obesity and adverse pregnancy outcomes [2–4]. Specifically, obesity increases the risk of miscarriage, gestational diabetes, hypertensive complications, infections, postterm pregnancy, and Cesarean section delivery [5–9]. Additionally, obesity is associated with numerous fetal risks including congenital anomalies, macrosomia, and perinatal morbidity and mortality [6, 7, 10–12]. However, it remains unclear whether obesity by itself, independent of diabetes and hypertension, is a predictor of adverse pregnancy outcome. A number of studies have provided indirect data suggesting a possible causal association between maternal adiposity and pregnancy complications. Endocrine abnormalities associated with obesity, that is, increased levels of insulin, androgens, and leptin, have been incriminated [13–16]. In fact, adipose tissue appears to be an active endocrine organ that secretes several proinflammatory cytokines, which may result in endothelial dysfunction in the mother and placenta and ultimately lead to adverse pregnancy outcome [13–16]. Challier et al. [13] have demonstrated that obesity in pregnancy results in an exaggerated inflammatory response in the placenta with accumulation of multiple subsets of macrophages and production of proinflammatory mediators. We hypothesize that the inflammatory and altered endocrine milieu in which the fetus develops may have detrimental consequences on fetal well-being. In this study, we investigate the impact of obesity on fetal well-being, as assessed by fetal blood gas parameters (BGPs), in a retrospective cohort of glucose-tolerant and nonhypertensive women. To the best of our knowledge, this is the first systematic assessment of the impact of obesity per se on umbilical artery BGP. 

## 2. Material and Methods

An Institutional Review Board approval was obtained prior to data collection. We searched our prospectively maintained database for all patients admitted to the labor and delivery department in our institution between January, 2011 and July, 2011. Patients with comorbidities (diabetes or hypertension) or conditions that could influence fetal BGP were systematically excluded from the analysis. Exclusion criteria were as follows: hypertension or pregnancy-induced hypertension; preeclampsia; diabetes, gestational diabetes or impaired glucose tolerance; active smoking or drug use; any medical comorbidity including congestive heart failure, chronic kidney disease, or chronic respiratory disease; placental abruption; chorioamniointis; nonreassuring fetal heart rate patterns; emergency Cesarean section delivery; vacuum extraction delivery; shoulder dystocia; multiple pregnancy; preterm birth; and congenital abnormality. Because the study was designed to compare obese to normal weight patients with regard to BGP, overweight patients (BMI 25–29.9 kg/m^2^) were excluded from the analysis.

Medical charts and laboratory results were carefully reviewed to determine patient's age, race, BMI, gravity, parity, number of prenatal visits, gestational age at delivery, mode of delivery, number of previous Cesarean section deliveries, group B streptococcal colonization, umbilical artery pH, PO_2_, PCO_2_ and base deficit, and Apgar score at 1 and 5 minutes. Patients were divided into 2 groups based on their prepregnancy BMI as follows: normal weight (<25 kg/m^2^, *n* = 50) and obese (≥30 kg/m^2^, *n* = 50). Umbilical artery blood gas analysis was performed at delivery. GBS colonization was marked as positive if any of the vaginal, anal, or urine cultures were positive at anytime. 

### 2.1. Statistical Analysis

A comparative analysis of BGP between the normal weight group and the obese group was conducted. Data are presented as mean and standard deviation for continuous variables and as frequency for categorical variables. Statistical analysis of categorical variables was carried out using chi-square and Fisher's exact tests as appropriate. Comparison of means was carried out using Student's *t*-test. A Pearson's correlation coefficient (*r*) calculation was also performed. *P* values of <0.05 were considered statistically significant. This study would have 99% power to detect a mean difference of 10 in base excess (type I error 5%) and 70% power to detect a mean difference of 5 (type I error 5%). All Statistical analyses including sample size calculation were performed usingSPSS(IBM Corporation, Armonk, NY, USA). 

## 3. Results 

A total of 100 patients (50 lean and 50 obese) were included in the study. BMI was 20.9 ± 2.1 kg/m^2^ on average in the normal weight group and 35.7 ± 5.0 kg/m^2^ in the obese group. There were no significant differences between the 2 groups with respect to all baseline characteristics except for parity (Table 1). Mean age was 25 ± 6.9 years in the normal weight group versus 26.9 ± 6.6 years in the obese group (*P* = 0.25). Hispanic patients accounted for 70% (*n* = 35) of the normal weight group versus 66% (*n* = 33) of the obese group (*P* = 0.67). A Cesarean section was performed in 22% (*n* = 11) of normal BMI patients compared to 28% (*n* = 14) of obese patients (*P* = 0.49). Eight (16%) lean patients and 9 (18%) obese patients tested positive for GBS (*P* = 0.79). 

There were no significant differences in any of the umbilical artery BGP between the two groups. pH and PCO_2_ were, respectively, 7.26 ± 0.06 and 54.61 ± 10.3 mmHg on average in the normal weight group versus 7.26 ± 0.08 (*P* = 0.95) and 55.12 ± 13.9 mmHg (*P* = 0.83) in the obese group. Mean umbilical artery PO2 was 25.7 ± 9.8 mmHg in lean patients versus 23.7 ± 8.9 mmHg in obese patients (*P* = 0.48). Newborns of lean patients had a 3.74 ± 1.8 mmol/L base deficit on average compared with 3.77 ± 2.4 mmol/L for newborns of obese patients (*P* = 0.79). Mean Apgar scores at 1 and 5 minutes were, respectively, 8.78 ± 0.5 and 9.08 ± 0.4 for newborns of lean patients versus 8.72 ± 0.6 (*P* = 0.62) and 9.14 ± 0.4 (*P* = 0.46) for newborns of obese patients. Similarly, Pearson's correlation coefficient did not reveal any significant correlation between BMI and BGP or Apgar scores (Table 2).

## 4. Discussion

 It is thought that obesity increases the risk of maternal and fetal complications mainly through its association with diabetes, hypertension, and other comorbid conditions. Thus, we excluded these potentially confounding factors from the analysis and investigated the impact of obesity per se on fetal well-being, namely, umbilical artery BGP and Apgar scores. Furthermore, because overweight is regarded as an intermediate state between normal weight and obesity, such patients were excluded from the analysis. Despite a well-matched study population for baseline characteristics, we did not find any significant difference between obese and lean patients with respect to BGP or Apgar scores. Our results suggest that obesity may not affect fetal well-being in patients with no comorbidities such as gestational diabetes or hypertension.

Only a few studies have investigated the association between obesity per se and pregnancy outcome. In a recent large study, Owens and colleagues [17] examined the impact of obesity on pregnancy outcome in 2329 glucose-tolerant women. They found an increased incidence of Cesarean deliveries, hypertensive complications, miscarriages, macrosomia, shoulder dystocia, and congenital malformations in obese women as compared to normal weight women. However, the authors did not assess the impact of BMI on umbilical artery BGP. Moreover, patients with hypertension or gestational hypertension were not excluded from the analysis which could have introduced bias into the study, especially given the established association between obesity and hypertension-related pregnancy complications [18]. In another large study, Jensen and colleagues [19] assessed the relationship between prepregnancy BMI and pregnancy outcome in 2459 glucose-tolerant Danish women. The risk of Cesarean section, induction of labor, hypertensive complications, and macrosomia was significantly increased in obese women compared with normal weight women. More importantly, however, the frequencies of shoulder dystocia, preterm delivery, and infant morbidity other than macrosomia were not significantly associated with maternal BMI. These findings are in line with the results of our study and suggest that obesity may not increase infant morbidity in the absence of diabetes or other comorbidities. The effect of obesity on umbilical artery BGP (among other variables) was investigated by Rode and colleagues [20] in a cohort of 8092 Danish women. The authors reported no difference between obese and lean patients with regard to umbilical pH or Apgar scores. Although the results of this study are in line with our findings, patients with diabetes/impaired glucose tolerance or hypertension were not excluded from the analysis which precludes any confident conclusion as to the impact of obesity, as an independent factor, on BGP and Apgar scores.

The limitations of this study stem primarily from its retrospective design, small sample size, and the absence of randomization. Although patients with nonreassuring fetal heart rate patterns (category III tracing) and vacuum extraction delivery were excluded, the influence of labor on the acid base status of the newborn may not have been completely controlled for in this study.

## 5. Conclusion

We found no relation between maternal BMI per se and umbilical artery BGP in a retrospective cohort of 100 patients. Thus, obese women who are glucose-tolerant and nonhypertensive may not be at increased risk of perinatal morbidity. Larger prospective studies are needed to confirm the absence of association between obesity and fetal well-being parameters. 

## Figures and Tables

**Table 1:** Baseline characteristics of normal weight and obese patients.

Baseline characteristics	Normal weight	Obese	*P* value
Age (years)	25.0 ± 6.9	26.9 ± 6.6	0.25*
Hispanics	35 (70%)	33 (66%)	0.67**
Gravity (mean)			
1	16 (32%)	8 (16%)	0.061**
2	9 (18%)	10 (20%)	0.80**
3	12 (24%)	15 (30%)	0.50**
4 or more	13 (26%)	17 (34%)	0.38**
Parity (mean)			
0	25 (50%)	15 (30%)	0.04**
1	14 (28%)	16 (32%)	0.66**
2	8 (16%)	8 (16%)	1.00**
3 or more	3 (6%)	11 (22%)	0.02**
Mean gestational age at birth (weeks and days)	38 and 6	39 and 1	0.14*
Cesarean section	11 (22%)	14 (28%)	0.49**
Previous Cesarean section	6 (12%)	8 (16%)	0.56**
Prenatal visits (mean)	9.73 ± 4.0	10.6 ± 3.3	0.26*
GBS	8 (16%)	9 (18%)	0.79**

Total	50	50	

*Student'S *t*-test, **chi-square test.

**Table 2:** Correlation of BMI with different variables.

	BMI
Mode of delivery	
Pearson's *r* coefficient	0.086
2-tailed *P* value	0.394
Previous Cesarean section	
Pearson's *r* coefficient	0.000
2-tailed *P* value	0.999
Number of visits	
Pearson's *r* coefficient	0.069
2-tailed *P* value	0.518
Race	
Pearson's *r* coefficient	−0.088
2-tailed *P* value	0.384
GBS	
Pearson's *r* coefficient	0.070
2-tailed *P* value	0.505
Gravity	
Pearson's *r* coefficient	0.089
2-tailed *P* value	0.381
Parity	
Pearson's *r* coefficient	0.011
2-tailed *P* value	0.913
pH	
Pearson's *r* coefficient	−0.010
2-tailed *P* value	0.924
Base deficit	
Pearson's *r* coefficient	−0.144
2-tailed *P* value	0.156
PO_2_	
Pearson's *r* coefficient	−0.100
2-tailed *P* value	0.322
PCO_2_	
Pearson's *r* coefficient	−0.023
2-tailed *P* value	0.820
Apgar 1 min	
Pearson's *r* coefficient	−0.025
2-tailed *P* value	0.807
Apgar 5 min	
Pearson's *r* coefficient	0.063
2-tailed *P* value	0.535
